# Barriers and Facilitators for Implementation of a Computerized Clinical Decision Support System in Lung Cancer Multidisciplinary Team Meetings—A Qualitative Assessment

**DOI:** 10.3390/biology10010009

**Published:** 2020-12-25

**Authors:** Sosse E. Klarenbeek, Olga C. J. Schuurbiers-Siebers, Michel M. van den Heuvel, Mathias Prokop, Marcia Tummers

**Affiliations:** 1 Department of Medical Imaging, Radboud Institute for Health Sciences, Radboud University Medical Center, P.O. Box 9101, 6500 HB Nijmegen, The Netherlands; Mathias.Prokop@radboudumc.nl; 2 Department of Pulmonary Diseases, Radboud Institute for Molecular Life Sciences, Radboud Institute for Health Sciences, Radboud University Medical Center, 6500 HB Nijmegen, The Netherlands; Olga.Schuurbiers-Siebers@radboudumc.nl (O.C.J.S.-S.); Michel.vandenHeuvel@radboudumc.nl (M.M.v.d.H.); 3 Department for Health Evidence, Radboud Institute for Health Sciences, Radboud University Medical Center, 6500 HB Nijmegen, The Netherlands; Marcia.Tummers@radboudumc.nl

**Keywords:** clinical decision support systems, oncology, pre-implementation, multidisciplinary team meetings, qualitative research

## Abstract

**Simple Summary:**

Oncological computerized clinical decision support systems (CCDSSs) are currently being developed to facilitate workflows of multidisciplinary team meetings (MDTMs). To successfully implement these systems in MDTMs, the aim of this qualitative assessment was to identify barriers and facilitators for implementation and to provide actionable findings for an implementation strategy. The main facilitators for implementation of the CCDSS were considered to be easy access to well-structured data, and reducing time spent by clinicians on MDTM preparation and duration of the MDTMs. Main barriers for adoption were seen in incomplete or non-trustworthy output generated by the system and insufficient adaptability of the system to local and contextual needs. Actionable findings for an implementation strategy were a usability test and validation study involving key users in the organization’s real-life setting. Given the growing interest in CCDSSs in oncology care, insight in barriers and facilitators for successful implementation seems highly relevant.

**Abstract:**

Background: Oncological computerized clinical decision support systems (CCDSSs) to facilitate workflows of multidisciplinary team meetings (MDTMs) are currently being developed. To successfully implement these CCDSSs in MDTMs, this study aims to: (a) identify barriers and facilitators for implementation for the use case of lung cancer; and (b) provide actionable findings for an implementation strategy. Methods: The Consolidated Framework for Implementation Science was used to create an interview protocol and to analyze the results. Semi-structured interviews were conducted among various health care professionals involved in MDTMs. The transcripts were analyzed using a thematic analysis following a deductive approach. Results: Twenty-six professionals participated in the interviews. The main facilitators for implementation of the CCDSS were considered to be easy access to well-structured patient data, and the resulting reduction of MDTM preparation time and of duration of MDTMs. Main barriers for adoption were seen in incomplete or non-trustworthy output generated by the system and insufficient adaptability of the system to local and contextual needs. Conclusion: Using a CCDSS in lung cancer MDTMs was expected to increase efficiency of workflows. Successful implementation was seen as dependent on the reliability and adaptability of the CCDSS and involvement of key users in the implementation process.

## 1. Introduction

Cancer is the second leading cause of death globally, and is responsible for an estimated 9.6 million deaths in 2018 [1]. The diagnosis and treatment of cancer patients is a complex, and rapidly evolving science. With increasing therapeutic options, the number of different health care disciplines involved in treatment is also expanding [2]. To ensure optimal patient care, multidisciplinary team meetings (MDTMs) have been introduced for clinical decision-making [3,4]. MDTMs have the goal to facilitate the delivery of high-quality cancer services and to improve guideline adherence as well as personalized treatment, ideally resulting in an improved quality of life and survival [3]. However, MDTMs often do not achieve their full potential [3]. Several challenges for MDTM workflows have been identified, such as lack of common standards, volume of data that needs to be processed, complexity of patients, tight time schedules and rapid development of diagnostic and therapeutic options [5]. As a consequence, variations in clinical workflows of MDTMs are experienced which might impact the quality of care for cancer patients [5,6,7].

In response to these challenges, a prototype oncological computerized clinical decision support system (CCDSS) for the use case lung cancer is currently being developed to assist health care providers in clinical decision-making. This oncological CCDSS is an active knowledge system that integrates multiple decision variables to support high quality of care and compromises of three main components. The first component provides a structured overview of patient variables that are essential for clinical decision-making, including findings from radiology, pathology, molecular diagnostics, laboratory tests, functional tests and patient characteristics. The second component links the individual patient information with clinical practice guidelines. This makes it possible to generate case-specific guideline-based recommendations about staging, diagnostic and stage-appropriate therapeutic options. Lastly, an analytics component provides hospitals insight in key performance indicators of their services. This approach enhances the availability and visualization of case-relevant information for multidisciplinary clinical judgement.

In the past, several reviews evaluated the impact of clinical decision support systems (CDSSs) across a variety of clinical settings [8,9,10,11,12]. Some reported improvements in patient care processes, clinician’s workflows, health care costs, use of preventative medicine and adherence to guidelines or standards of medical practice [8,9,10,11]. Others found no effect or negative effects [10,11,12]. Research has attempted to pinpoint causes of these mixed results: as one of the primary reasons it identified poor adoption of the systems by clinicians due to lack of willingness or ability to use technological systems [13,14,15,16]. As many designs of CDSSs have not incorporated input from clinicians during development, clinicians have a low level of experience and do not always trust the quality of the systems’ algorithms and the output generated [13,14,15,16]. Consequently, clinicians hesitate to accept CDSSs, leading to suboptimal implementation [15]. 

Engagement and involvement of key users in early stages of implementation is highly important to reach successful implementation and avoid low clinical adoption. Therefore, a qualitative study, guided by the Consolidated Framework for Implementation Research (CFIR), was conducted to get an in-depth understanding of the attitudes, motivations, individual preferences and clinical perspectives regarding the CCDSS from diverse medical disciplines involved in lung cancer MDTMs [17]. The research objectives were to identify barriers and facilitators for successful implementation of the oncological CCDSS for the use case of lung cancer; and to provide actionable findings for an implementation strategy.

## 2. Methods

This study was conducted as part of a research project involving the development, implementation and evaluation of an oncological CCDSS for the use case of lung cancer.

### 2.1. Study Design

A multicenter qualitative study was conducted among health care professionals involved in lung cancer care in various Dutch hospitals. A semi-structured interactive interview method was used to answer the research question. First, interviews were initiated at the academic hospital that will be first to implement and deploy the CCDSS. Second, stakeholders from other hospitals were approached to verify the results. All hospitals that were contacted for participation have a focus and specialization on lung cancer care. 

### 2.2. Study Participants—Sampling and Recruitment

According to the MDTM quality criteria of the Dutch national cancer registry, presence of a core team containing the following medical disciplines is required: surgeon, pulmonologist, radiologist, pathologist, radiotherapist, case manager and nursing specialist [18]. Stakeholders of these disciplines were selected and approached for an interview. To account for variability in answers, multiple specialists per discipline were contacted for participation. Table 1 shows the reasons for inclusion of each discipline. Snowballing or referral sampling techniques were used to contact and interview other relevant stakeholders. In accordance with local ethical guidelines, no ethical approval was required due to the professional nature of the study participants. All stakeholders received information about the aim of the study and gave written informed consent before participation. Sampling was discontinued when saturation of information was achieved.

### 2.3. Data Collection-Methods

Data was collected using semi-structured interactive interviews guided by the interview protocol (Supplementary Material S1: Interview protocol). The interactive character of the interviews meant that questions could be adjusted or added based on the information gained during earlier interviews. In addition, the views of earlier participants were discussed anonymously during interviews with the next participants to gain an in-depth understanding of the key statements. All interviews have been conducted by the first author (SK), who was trained by an expert on qualitative research (MT) and received weekly supervision between May and December 2019. The majority of the interviews took place in person at a location preferred by the participants. Two participants were interviewed by use of videoconferencing and three by phone. The interviews had an average duration of 50–60 min and were recorded with a voice recorder app. 

### 2.4. Data Collection-Instrument

An interview protocol was created based on the CFIR and expert opinion (Supplementary Material S1: Interview protocol). Damschroder et al. developed the CFIR that offers an overarching typology to promote implementation theory development and verification about what works where and why across multiple contexts [17]. The CFIR consists of a compilation of 39 constructs divided into five domains: characteristics of the intervention (CCDSS), the inner setting (lung cancer MDTMs, involved departments and workflows), the outer setting (the context in which MDTMs resides), individual characteristics (the MDTM core team of specialists), and the process used for implementation of the intervention. The interview was started with an explanation of the purpose of the interview and the goals. During the interview the three main components of the CCDSS were introduced by the interviewer: (1) structured overview of patient variables, (2) linking individual patient information with clinical practice guidelines, and (3) an analytics component. The interview protocol contained open-ended questions that focused on the following topics: (a) participants’ perspective on current MDTMs, (b) need for change in current MDTMs, (c) interpretation of the CCDSS and (d) identification of barriers and facilitators for implementation of the CCDSS. 

### 2.5. Data Analysis 

**Figure 1:**
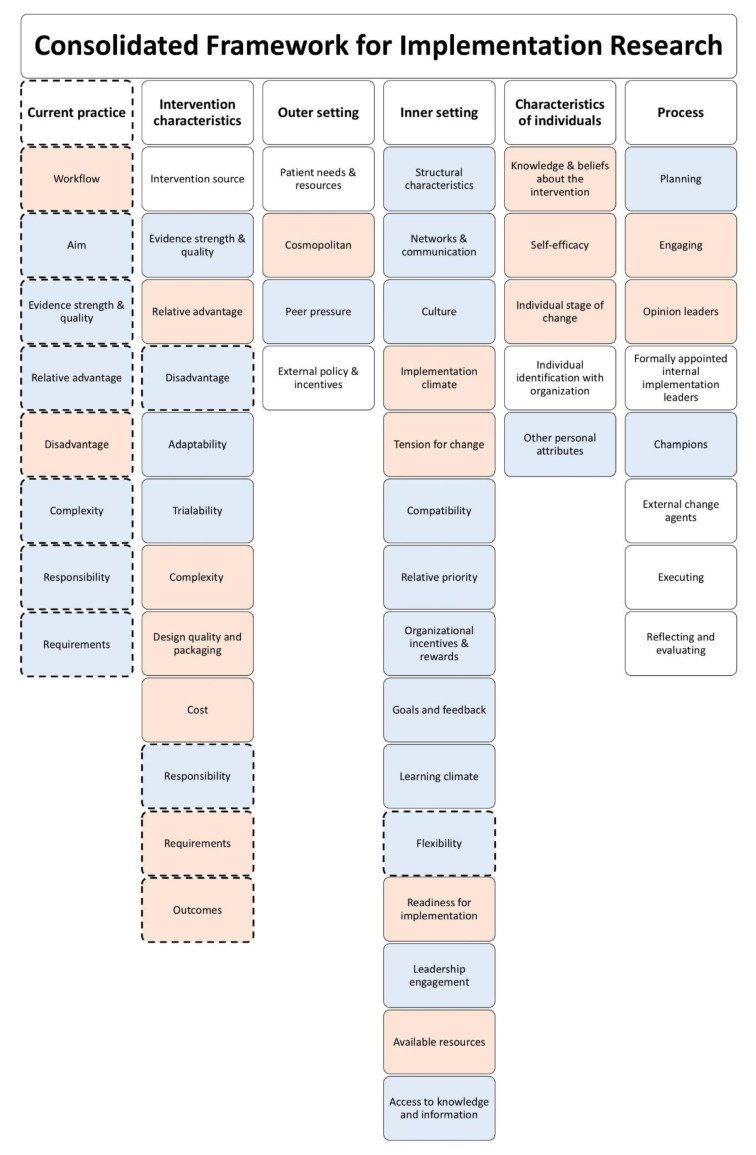
Overview of the elements of the final codebook and the components taken from the Consolidated Framework for Implementation Research (CFIR). The dotted boxes represent the domain and constructs added to the original CFIR codebook during analysis. The blue and orange boxes represent constructs that were initially captured by the interviewer during phase 2 of thematic analysis (44/53). The orange boxes represent barriers and facilitators identified as relevant for answering the research question (18/53). The blue boxes represent barriers and facilitators that were interesting but not necessary for answering the research question.

The transcripts of the interviews were analyzed using thematic analysis in a stepwise manner as described by Braun and Clark following a deductive approach [19]. Thematic analysis consists of six phases: (1) *Familiarising yourself with the data*: Audio recordings of the interviews were transcribed verbatim, and a summary of each interview was sent to the participant for checking the content. (2) *Generating initial codes*: Initial codes were created with use of the CFIR domains and constructs. Transcripts were coded using Atlas.ti Scientific Software Development GmbH, Berlin, version 8.4.20. After the first author (SK) coded two transcripts, those were reviewed by the last author (MT). New codes were created as some parts of the transcripts contained information that was not covered by any of the CFIR constructs. The final codebook can be found in Supplementary Materials S2 (Table S2: Codebook). (3) *Collating codes into potential themes*: As a deductive approach was followed, the primary task in this phase was to resort and re-evaluate the codes. (4) *Reviewing themes*: All themes and codes were reviewed and if necessary reconsidered (SK and MT). (5) *Defining and naming themes*: Constructs represented by the orange and blue boxes in Figure 1 represent the elements of the final codebook that were captured initially by the interviews. Constructs represented by the orange boxes were identified as relevant to answer the research question. The blue boxes represent constructs that were interesting but not necessary to answer the research question. (6) *Producing the report*: Representative quotes were selected that best represented views and findings on the various themes. No distinction has been made in the data analysis and result section between answers by professionals from the various hospitals.

## 3. Results

### 3.1. Demographics

Table 2 provides an overview of the 26 professionals who participated in this qualitative assessment. Twelve of the professionals were female (46%), 14 were aged 40–60 years (54%) and 24 worked at an academic hospital (92%).

### 3.2. Identification of Potential Barriers and Facilitators

Potential barriers and facilitators identified as relevant for answering the research question were sorted according to the five domains of the CFIR and summarized in Table 3. Constructs considered irrelevant were left out. We added the domain current practice to be able to cover professionals’ perspectives on current clinical practice and current MDTMs. To illustrate the main points, we provided quotes of professionals, literally transcribed from the interviews and linked with the professional ID. Brackets are placed around words inserted to improve readability of the quotes. 

### 3.3. Current Practice—MDTMs

The majority of the professionals experienced current MDTM workflows as time-consuming. An important reason was that patient data relevant for MDTMs is not consistently stored at specified locations in the electronic medical record (EMR) but at various locations. This makes it difficult to find and retrieve all relevant patient information. Reasons are, for example, an inefficient user interface but also unstructured clinical reporting by clinicians, and storage of patient data in scanned documents or external systems. This was expected to form a barrier for accurate functioning of the CCDSS, if the CCDSS is not able to recognize and locate all data essential for decision-making. A structured way of working was seen as beneficial for accurate functioning of the CCDSS. Components of a structed way of working include consistent use of terminology by all medical disciplines and storage of information at dedicated locations within the EMR, including retrieval of information from scanned or external documents.

[Professional ID: 4] *‘Frequently, clinicians have to search for additional patient data in the EMR during MDTMs. This takes time as the information is often stored in clinical notes and therefore cannot be easily retrieved*.*’*

### 3.4. Intervention Characteristics—CCDSS

Professionals suggested that if the CCDSS was able to provide easily accessible and well-structured patient data, this would enable a more to-the-point, structured, standardized and transparent MDTM discussion. Well-structured patient data was defined as a clear customized patient-record consisting of discrete data fields with standardized response of parameters (e.g., age, weight, stage) that can be directly incorporated in clinical decision-making. Therefore, implementation of the CCDSS was expected to reduce the time necessary for MDTM preparation and to shorten the duration of MDTMs. In addition, it would reduce administrative burden. Less impact was expected on the quality and accuracy of decisions taken during the MDTM and on clinical outcomes because the professionals believed that MDTMs already facilitated high-quality lung cancer services due to the presence of extensive medical expertise and knowledge. Compared with existing EMR tools, the CCDSS would be able to offer insight into whether the data essential for decision-making was complete. This was seen as a facilitator for using the system as it provided guidance which patients should be discussed at the MDTM, reducing time and effort wasted on patients of which crucial information is missing.

[Professional ID: 17] *‘If the system provides a summarized and structured overview with all relevant patient variables, we do not have to extract those data manually from clinical notes and medical letters anymore. This will save us lots of time.’*

[Professional ID: 4] *‘After a patient case is introduced during the MDTM, it frequently becomes apparent that key diagnostic reports are not available yet. Time and effort is wasted on preparation and introduction for clinicians involved as these patients have to be discussed again in the next MDTM.’*

When professionals were asked how the CCDSS could fit into existing MDTM workflows, they requested a user-interface that was easy and intuitive with good readability of the data displayed by the system. Professionals also stated that the output must be correct, complete and up-to-date as the output serves as basis for MDTM decision-making. This was seen as challenging because of rapid evolution in diagnostic and therapeutic options in lung cancer care. Other potential obstacles for uptake were implementing an extra clinical system on top of the EMR and high purchase costs. Further, with the introduction of the second component of the CCDSS, professionals felt legally more vulnerable for potential criticism from patients since the system would expose discrepancies between formal guidelines and contextualized decisions. 

### 3.5. Outer Setting

Included hospitals had close collaborations with other hospitals. Meaning that if a second opinion was requested by one of the other hospitals, patients were discussed by use of video-conferences during MDTMs. Therefore, it is necessary to involve external partners in the implementation process in order to make sure the CCDSS could be adapted to all types of commercial EMRs. 

### 3.6. Inner Setting

Professionals expressed need for change in current MDTM workflows. They also expected that most of those required changes could be addressed by the CCDSS. Besides the already mentioned MDTM preparation time and total MDTM duration, professionals are in need of methods to discuss complex and non-complex patients, to visualize pathology slides and to improve MDTM reporting. In addition, the majority of professionals believed limited additional resources and technical facilities were necessary to facilitate implementation of the CCDSS.

[Professional ID: 6] *‘The MDT report has to reflect the content of the MDT discussion. This is often not the case as essential details are not reported. Consequently, certain aspects need to be discussed again, making this process very inefficient.’*

### 3.7. Characteristics of Individuals

Not all components of the CCDSS were valued the same way by all professionals. For example, pulmonologists expected the second component (linking individual patient information with clinical practice guidelines) to have low impact on MDTM workflow and decision-making because Dutch national lung cancer guidelines were frequently outdated due to rapid development of new drug treatments. They prioritized development and implementation of the first component (structured overview of patient variables) since they believed output of this component would serve as basis for decision-making. Some professionals expressed concerns regarding the reliability of the output generated by the CCDSS because of scepticism about the accuracy of the system’s algorithms. They also questioned the adaptability of the system to vast diversity of clinical presentations and the impact of structured patient data on quality of decision-making as clinical nuances (e.g., psychosocial determinants) potentially lack in these templates.

[Professional ID: 19] *‘I do not know how variables are defined and processed by the system. How reliable is this? Who is going to check all these data?’*

In attempt to overcome these barriers of implementation, all professionals suggested to perform a usability test and validation period for the prototype CCDSS in the organization’s real-life setting prior to roll-out to test performance. Further, most professionals expressed willingness to change current workflows in order to benefit from the CCDSS.

[Professional ID:10] ‘*We can schedule some extra time before each MDTM in order to discuss a couple of patients with use of the system. Straightforward cases are preferred.’*

### 3.8. Process

The professionals were asked to share their thoughts about how to organize successful implementation of the CCDSS. In order to actively engage end users, they emphasized the importance to inform clinicians about the added value that the system will bring to clinical practice. Therefore, the purpose and benefits of the system should be made very clear in contextual activities, such as an introduction, training or e-learning. Other suggestions to increase uptake of the system were involvement of clinicians from multiple disciplines in the further development of the system and implementation, and creating opportunities for users to become familiar with the CCDSS.

The majority of the professionals identified pulmonologists and nursing specialists in pulmonary oncology as key opinion leaders, as their support was perceived as essential for successful implementation in clinical practice. Those opinion leaders could provide input on the systems’ design and define parameters essential for MDTM decision-making. Pulmonologists were seen as superusers who could lead the implementation process and could inspire and encourage other disciplines to use the system. A few professionals also highlighted involvement of management teams in the implementation process because of their responsibility for financial and contractual agreements with suppliers.

[Professional ID:6] *‘Involve the managing directors of all involved medical departments. I assume that due to the system, these departments will get an additional financial burden. To ensure back-up from the management, you must be able to provide insight in these costs.’*

## 4. Discussion

### 4.1. Summary of Evidence

Key users were interviewed in this study to identify barriers and facilitators for successful implementation of an oncological CCDSS in lung cancer MDTMs, guided by the CFIR framework. The CCDSS was perceived as a helpful information tool at the service of clinicians in order to achieve the full potential of MDTMs. The main facilitators for implementation were considered to be easy access to well-structured patient data, and the resulting reduction of MDTM preparation time and duration of MDTMs. Main barriers for the adoption included potentially incomplete or non-trustworthy output generated by the system and insufficient adaptability of the system to local and contextual needs. To overcome barriers for implementation, key users suggested to perform a usability test involving key users and validation in the organization’s real-life setting prior to roll-out.

### 4.2. Implication of Results

Our study contributes to the literature in three main ways. Beginning, we were first to explore possibilities to utilize oncological CCDSSs in MDTMs. Introduction of oncological CCDSSs in MDTMs is not just an off-on switch as this complex clinical setting is in need for an implementation strategy that goes beyond an approach focused on optimizing CCDSSs’ usability and validity. Factors as complex clinical workflows, multiple clinical preferences and perspectives, clinicians’ attitudes towards scientific evidence, outdated national lung cancer guidelines and methods for collaboration with product developer and other players in the CCDSS landscape should be addressed. Second, this study showed the importance of involving end users in early stages of development and implementation as the more clinicians gain a sense of control over a system like the CCDSS, the more likely they are to perceive the technology as a valuable working tool that can complement their competencies and skills. This brings us to the third contribution of our study: with the use of the identified barriers and facilitators we have defined four actionable findings that should be addressed in an implementation strategy: (1) Perform a usability test and validation of the prototype CCDSS in real-life setting prior to implementation; (2) Demonstrate the purpose and potential benefits of the CCDSS to end users and create opportunities to familiarize themselves with the system; (3) Involve external partners and end users in further development and implementation and actively monitor and evaluate this involvement; (4) Actively engage pulmonologists, nursing specialists and management of the involved medical departments.

### 4.3. Relation to Other Studies

The professionals expected by the first component of the CCDSS, structured overview of patient variables, to increase efficiency in MDTM workflows (CFIR: Intervention Characteristics). The studies of Khalifa and Sutton et al. described similar findings as they reported that the use of decision support systems in clinical practice were expected to minimize the time spend by clinicians on manual data entry, which increased efficiency but also ensured time is not being taken away from direct patient care [20,21]. The literature overview of Shahsavarani et al. confirmed time saving to be the most important benefit of using decision support systems in clinical settings [22]. This study included 27 articles and concluded that decision support systems were expected to increase effectiveness and efficiency in clinical workflows by providing useful and critical information to desirable decision-making, faster process of orders, reduction of percentage of repeated medical tests, reduction of drug side-effects and change in drug consumption patterns [22]. The study of Verberne et al. confirmed this expected time saving in clinical workflows for the follow-up of colorectal cancer: in their clinical study reported a significant reduction in clinician’s workload in the from 64 min to 23 min per patient per year after implementation of a CCDSS [23]. This, however, is the only study that verified these assumptions. Further, a clear and intuitive user interface was suggested to increase uptake (CFIR: Intervention Characteristics). Castillo et al. confirmed emphasis should be placed on creating a simple display as this allows users to work efficiently without compromising workflows [24]. 

Professionals raised concerns about reliability of the output generated by the system and the adaptability of the system to local and contextual needs (CFIR: Characteristics of Individuals). Previous research confirmed that frequently found errors in data processing and output of clinical decision support tools negatively affect users’ perception as unsuitable suggestions for decision-making lead to doubts about safety and accuracy [25,26,27]. Resistance was also expressed regarding the potential lack of clinical nuances for psychosocial factors in the well-structured patient overview provided by the first component (CFIR: Characteristics of Individuals). The predominant role of psychosocial information in MDTM decision-making is supported by Horlait et al. as failure to account for this information negatively impacted the implementation of treatment recommendations formulated during MDTMs [28]. However, difficulties were described by Senteio et al. regarding the ability to capture psychosocial information in discrete data fields [29]. Professionals emphasized the need for information standards that provide the CCDSS the ability to capture and standardize psychosocial determinants (e.g., social economic status, social support, mental health). Despite the CCDSS enhancing case-relevant information for lung cancer staging and treatment selection, professionals doubted if the MDTM workflows would benefit from this system (CFIR: Characteristics of Individuals). However, previous literature suggested room for improvement in care efficacy of lung cancer MDTMS as 16% of patients failed to achieve prompt MDTM treatment decisions, mainly caused by further investigations and insufficient pathology [30,31,32]. Recently, initial evidence was reported for the potential of CDSSs in lung tumor boards as using a CDSS enabled clinicians to get to higher quality decisions compared to their normal way of working (median = 3.75 out of 5; min = 3 and max = 4) [33]. However, as they evaluated only eight primary lung cancer cases, evidence regarding the real impact of CDSSs on MDTM parameters remains scarce. As professionals expected the CCDSS to provide insight into whether data essential for MDTM decision-making is complete (CFIR: Intervention characteristics), it would be worthwhile to assess its impact on MDTM care efficacy as this potentially shortens the interval from diagnosis to treatment and increases survival and quality of life [32]. Performing a usability test and validation prior to roll-out was highly prioritized by most professionals to become familiar with the system and to evaluate its performance (CFIR: Characteristics of Individuals, Process). The study of Khairat et al. confirmed that prototypic decision support systems should undergo a rigorous usability test in a real-life setting to ensure performance of the system and to emphasize clinicians’ trust in the data and output processed by the system [15]. The study of Press et al. emphasized the need for usability testing of complex clinical decision support tools as this allows optimization of a tool prior to its integration into the clinical workflow environment [34]. Methodologies documented as successful for testing the usability of clinical decision support tools in health informatics are ‘think-aloud’ protocols and ‘near-live’ simulations [35,36]. Both provide the ability to identify real-life barriers to adoption and surface level usability issues.

### 4.4. Strengths and Weaknesses of This Study

Our study has several limitations, starting with the most significant and therefore most important limitation: As our study was performed during the development phase of the system, the CCDSS was introduced verbally. Therefore, participants’ interpretation of the system may vary greatly as they commented only based on what they believe and not on actual experience. Added, professionals were selected based on the Dutch MDTM quality criteria. Factors such as age, seniority and affinity with digital innovations were not taken into consideration firsthand. However, these factors may affect the interpretation of the system considerably. For example, the residents that participated grew up in a more digitalized educational and working environment and may therefore intrinsically tend to more positive attitudes towards systems like the CCDSS when compared to senior specialists. It must be mentioned that identified barriers and facilitators do not necessarily have to correspond to barriers and facilitators that would be identified based on actual experience. Furthermore, two professionals have been interviewed by use of videoconference and three by phone. In-person interviews were preferred to be able to read and interpret body language when needed. Third, professionals only included end users of the CCDSS. Other actors that play an important role in shaping the structural and political support of CCDSSs’ adoption, such as information technology staff or managers, were not taken into consideration. Fourth, the information in qualitative research is not always obtained in the same manner due to inter-interview differences in communication. However, our use of an interview protocol attempted to standardize the process as much as possible. Finally, the study is limited in its generalizability as only professionals working at hospitals specialized in lung cancer care had been approached. To strengthen our findings, comparisons with studies conducted in organizational settings and other countries will be necessary.

Our study has several major strengths. First, using an existing framework within the field of implementation science is considered an important strength to better understand, describe and identify factors that are relevant for implementation. Second, a local identification of potential barriers and facilitators is provided as this study was performed in departments where implementation will take place. Another strength is reaching saturation within all domains overall, except for the domain process, as the system is still in the development phase. Lastly, the interactive nature of the interviews facilitated a more comprehensive insight in the perspectives of the stakeholders with regard to the CCDSS. Through verification of stakeholder claims with other stakeholders, validity was enhanced.

### 4.5. Future Research

A post-implementation study of the CCDSS could provide insight on how issues identified in the present study are being addressed and what challenges remain. The introduction and uptake of the CCDSS should also be examined longitudinally in order to analyze its long-term impact. This would be especially useful if the follow-up study could also cover the perspectives of other actors that play an important role in the implementation process.

## 5. Conclusions

Using this CCDSS in lung cancer MDTMs was expected to increase efficiency in MDTM workflows if the system provides easy access to well-structured patient data. Less impact was expected on the quality of lung cancer services generated by MDTM decision-making. Successful implementation was seen as dependent on the reliability and adaptability of the CCDSS and involvement of key users in the implementation process. A post-implementation interview study could provide insight in the remaining challenges. The findings of this study inform further decision strategies for successful implementation of oncological CCDSSs in MDTMs. 

## Figures and Tables

**Table 1:** Reasons to include MDTM stakeholders.

Stakeholder	Reason for Inclusion
Pulmonologist	Pulmonologists subscribe patients with a suspicion of lung cancer for MDTMs and play a central role in the diagnostic trajectory, therapeutic decision-making, and the administration of systemic treatment. Besides, they also chair the MDTM and have a key role in ensuring that this meeting proceeds at an appropriate pace to finish at the scheduled time.
Cardiothoracic surgeon	Cardiothoracic surgeons assess resectability of tumors and operability of patients.
Radiologist	Radiologists discuss and explain radiological findings and provide input for clinical staging.
Radiotherapist	Radiotherapists assess and discuss radiotherapeutic treatment options.
Nuclear medicine physician	Nuclear medicine physicians assess and interpret nuclear medicine and hybrid imaging results.
Pathologist	Pathologists discuss pathology results, interpret predictive biomarkers, and provide pathological staging of the disease.
Specialist nurses	Nurses provide input about the social and psychological condition of the patient, important in therapeutic decision-making.
MDTM secretary	Secretaries are involved in planning of the meetings and recording of results.

MDTMs: Multidisciplinary Team Meetings.

**Table 2:** Overview of the professionals.

Professional ID	Specialty	Hospital (Academic/General)	Gender (Female/Male)	Age Group (Years)	Seniority (Years)	Frequency of MDTM Visits
1	Radiologist in training	Academic	Male	20–40	Resident	Non-frequent
2	Pulmonologist	Academic	Male	41–60	18	Weekly
3	Cardiothoracic surgeon	Academic	Male	41–60	10	Weekly
4	Radiologist	Academic	Male	20–40	5	Weekly
5	Radiologist	Academic	Female	41–60	6	Weekly
6	Pulmonologist	Academic	Male	41–60	14	Weekly
7	Radiologist	Academic	Male	41–60	11	Weekly
8	Nuclear medicine physician	Academic	Male	20–40	4	Weekly
9	Pulmonologist	Academic	Female	41–60	15	Weekly
10	Radiologist	Academic	Female	41–60	12	Weekly
11	Nursing specialist pulmonary oncology	Academic	Female	20–40	20	Weekly
12	Radiologist	Academic	Male	41–60	11	Weekly
13	Radiation oncologist	Academic	Male	41–60	23	Weekly
14	Nursing specialist pulmonary oncology	Academic	Female	20–40	12	Weekly
15	Cardiothoracic surgeon	Academic	Male	41–60	23	Weekly
16	Secretary MDTM	Academic	Female	20–40	10	Monthly
17	Radiologist in training	Academic	Female	20–40	Resident	Non-frequent
18	Pathologist	Academic	Female	20–40	1	Weekly
19	Radiologist	Academic	Female	41–60	20	Weekly
20	Pulmonologist	Academic	Female	41–60	17	Weekly
21	Pathologist	Academic	Male	41–60	30	Weekly
22	Pulmonologist	Academic	Male	20–40	9	Weekly
23	Pathologist	Academic	Male	41–60	15	Weekly
24	Pulmonologist	General	Female	20–40	8	Weekly
25	Radiologist	Academic	Female	41–60	10	Weekly
26	Radiologist	General	Male	20–40	8	Weekly

MDTMs: Multidisciplinary Team Meetings.

**Table 3:** Potential barriers and facilitators for implementation of the oncological CCDSS for lung cancer MDTMs.

Domain and Construct	Potential Barriers	Potential Facilitators
Current practice		
Workflow		A structured way of working in clinical practice, e.g. use of consistent terminology by all medical specialists and disciplines and storage of information at dedicated locations within the EMR
Disadvantage	Inconsistent storage of relevant patient data at specified locations in the EMR, making it difficult for the CCDSS to recognize and locate all data essential for decision-making	
**Intervention characteristics**		
Relative advantage		Easily accessible and well-structured patient data
		Insight into whether data essential for MDTM decision-making is complete or entries are missing
Complexity	Implementation of an extra clinical system on top of an EMR	Intuitive user-interface
	Incomplete or incorrect output of the CCDSS	
	The CCDSS may expose discrepancies between formal guidelines and contextualized decisions, making professionals legally more vulnerable for criticism from patients	
Design quality and packaging	Poor readability of the data displayed by the CCDSS during MDTMs	
Costs	Unfavorable cost-benefit ratio	
Outcome		Reduced MDTM preparation time and duration of MDTMs
**Outer setting**		
Cosmopolitanism		Adaptability to various types of EMR
**Inner setting**		
Implementation climate		
Tension for change		Needs for change in current MDTMs: Preparation time Total duration of the meeting Methods to discuss complex and non-complex patients Reporting structure Visualization of pathology slides
Readiness for implementation		
Available resources		Limited additional resources and technical facilities during MDTMs
**Characteristics of individuals**		
Knowledge & beliefs	Dutch national lung cancer guidelines are considered outdated, which makes guideline adherence not important	Prioritize development and implementation of the first component (structured overview of patient variables) because this component was expected to improve MDTM workflows most
	Lack of trust among professionals in accuracy of system’s algorithms	Perform a usability test and validation of the prototype CCDSS in real-life setting prior to roll-out
Self-efficacy		Willingness of professionals to change current workflows in order to benefit from CCDSS
**Process**		
Engaging		Provide insight into purpose and potential benefits of the CCDSS
		Involve end users and external partners during development and implementation
		Create opportunities for users to familiarize themselves with the CCDSS
Opinion leaders	Lack of acceptance by key opinion leaders	Institute pulmonologists as superusers who lead implementation
		Engage management to arrange internal and external financial and contractual agreements

EMR: Electronic Medical Record, CCDSS: Computerized Clinical Decision Support System, MDTMs: Multidisciplinary Team Meetings.

## Data Availability

Data sharing is not applicable due to privacy of the study participants.

## Supplementary Materials

The following are available online at https://www.mdpi.com/2079-7737/10/1/9/s1. S1: Interview Protocol, S2: Codebook.

## Abbreviations

CDSS: Clinical Decision Support System; CCDSS: Computerized Clinical Decision Support System; CFIR: Consolidated Framework for Implementation Research; EMR: Electronic Medical Record; MDT: Multidisciplinary Team; MDTM: Multidisciplinary Team meeting.
