# Supplementary material for: Barriers and Facilitators for Implementation of a Computerized Clinical Decision Support System in Lung Cancer Multidisciplinary Team Meetings—A Qualitative Assessment
Source: Biology (Basel). 2020 Dec 25;10(1):9. doi: 10.3390/biology10010009 (PMC7830066; doi:10.3390/biology10010009)
Supplement: Supplementary file 1 [file biology-10-00009-s001.zip › biology-980645-supplementary/S1_InterviewProtocol.pdf]

**S1: Supplementary Material 1. Interview Protocol:**

**A. Vragen omtrent MDO long:**

1. <u>*Huidige situatie MDO long*</u>:

- Wat is uw rol binnen het long MDO en kunt u wat vertellen over deze rol? *(Subvraag: Wat vind u van deze rol?)*
- Wat is volgens u het overkoepelende doel van het long MDO? *(Subvragen: Wordt dit doel over het algemeen bereikt binnen het MDO? Waarom wel/niet? Hoe uit zich dit?)*
- Waar ziet u de grootste meerwaarde van het MDO? (Voor u als arts? Voor de patiënt?) *(Subvragen: Hoe uit zich dit? Welke meerwaarde is voor jou het meest van belang?)*

2. <u>*Ruimte voor verbetering:*</u>

- Wat gaat er volgens u goed binnen het long MDO? *(Subvragen: Waarom gaan specifiek deze aspecten goed? Hoe uit zich dit?)*
- Waar is er ruimte voor verbetering? *(Subvragen: Waarom juist op deze aspecten?)*
- Als we het hebben over de ruimte voor verbetering, zie u manieren hoe dit opgelost kan worden? *(Subvragen: Waarom op deze manier(en)? In welke concrete acties kunnen deze oplossingen/manieren omgezet worden?)*

**B. Vragen omtrent interventie en implementatie:**

*3.* <u>*Interventie:*</u>

- Wat is je eerste indruk van het systeem?
- Wat zou zo'n systeem kunnen toevoegen aan het long MDO? *(Subvraag: Waarom op deze aspecten?)*
- Welke effecten zou zo'n systeem kunnen bewerkstellingen buiten het MDO? (patiënt, planning van zorg, etc). *(Subvraag: Waarom deze effecten?)*
- Wat zou volgens u de hoofdfocus van zo'n systeem moeten zijn? *(Subvraag: Waarom?)*
- Wat vindt u van de eerder benoemde componenten van het systeem? *(Subvraag: Heeft u hierop nog toevoegingen?)*
- In hoeverre sluit zo'n systeem aan op knelpunten waar u binnen het MDO tegenaan loopt? *(Subvraag: Hoe zou het systeem beter kunnen aansluiten?)*
- Wat zijn voor u voorwaarden/criteria waar zo'n systeem tenminste aan moet voldoen voordat u deze zou willen inzetten binnen het MDO?

4. <u>*Implementatie*</u>:

- Hoe zou dit systeem volgens u een plek kunnen vinden binnen het MDO? *(Subvraag: Welke acties moeten hiervoor in gang gezet worden?)*
- Welke aanpassingen aan de huidige werkwijze vraagt implementatie van dit systeem? (Subvragen: *In hoeverre zijn deze aanpassingen mogelijk*? *In hoeverre is het MDO hierin flexibel?)*
- Wat zijn factoren die mogelijk de implementatie kunnen bevorderen of beperken?
- Als we kijken naar de bevorderende factoren, hoe kunnen we deze volgens u optimaal benutten?
- Als we kijken naar de beperkende factoren, hoe zou u deze factoren aanpakken om hindering zo laag mogelijk te houden?
- Binnen het MDO zijn verschillende disciplines betrokken, in hoeverre is dit van invloed op de implementatie van het systeem?
- Wat zouden voor u de eerste stappen zijn richting implementatie?

- Welke rol kunt u spelen in de implementatie van dit systeem?
